# High nuclear MSK1 is associated with longer survival in breast cancer patients

**DOI:** 10.1007/s00432-018-2579-7

**Published:** 2018-01-11

**Authors:** Xuan Pu, Sarah J. Storr, Narmeen S. Ahmad, Emad A. Rakha, Andrew R. Green, Ian O. Ellis, Stewart G. Martin

**Affiliations:** 1 University of Nottingham, Division of Cancer and Stem Cells, Department of Clinical Oncology, School of Medicine, Nottingham University Hospitals NHS Trust, City Hospital Campus, Nottingham, UK; 2 University of Nottingham, Division of Cancer and Stem Cells, Department of Histopathology, School of Medicine, Nottingham University Hospitals NHS Trust, City Hospital Campus, Nottingham, UK

**Keywords:** Breast cancer, MSK, Breast cancer-specific survival, Biomarker

## Abstract

**Purpose:**

Mitogen- and stress-activated kinases (MSKs) are important substrates of the mitogen-activated protein kinase (MAPK)-activated protein kinase family. MSK1 and MSK2 are both nuclear serine/threonine protein kinases, with MSK1 being suggested to potentially play a role in breast cancer cell proliferation, cell cycle progression, cell migration, invasion and tumour growth. The aim of the current study was to assess MSK1 protein expression in breast cancer tumour specimens, evaluating its prognostic significance.

**Methods:**

A large cohort of 1902 early stage invasive breast cancer patients was used to explore the expression of MSK1. Protein expression was examined using standard immunohistochemistry on tissue microarrays.

**Results:**

Low MSK1 protein expression was associated with younger age (*P* = 0.004), higher tumour grade (*P* < 0.001), higher Nottingham Prognostic Index scores (*P* = 0.007), negative ER (*P* < 0.001) and PR (*P* < 0.001) status, and with triple-negative (*P* < 0.001) and basal-like (*P* < 0.001) phenotypes. Low MSK1 protein expression was significantly associated with shorter time to distant metastasis (*P* < 0.001), and recurrence (*P* = 0.013) and early death due to breast cancer (*P* = 0.01). This association between high MSK1 expression and improved breast cancer-specific survival was observed in the whole cohort (*P* = 0.009) and in the HER2-negative and non-basal like tumours (*P* = 0.006 and *P* = 0.024, respectively). Multivariate analysis including other prognostic variables indicated that MSK1 is not an independent marker of outcome.

**Conclusions:**

High MSK1 is associated with improved breast cancer-specific survival in early stage invasive breast cancer patients, and has additional prognostic value in HER2-negative and non-basal like disease. Although not an independent marker of outcome, we believe such findings and significant associations with well-established negative prognostic factors (age, grade, Nottingham Prognostic Index, hormone receptor status, time to distant metastasis, recurrence and triple-negative/basal-like status) warrant further examination and validation in independent patient cohorts.

## Background

Mitogen- and stress-activated kinases (MSKs), nuclear serine/threonine protein kinases, are important substrates of the mitogen-activated protein kinase (MAPK)-activated protein kinase family [reviewed in (Cargnello and Roux 2011)]. Two novel protein kinases, MSK1 and MSK2, were originally described by Deak et al. in 1998, based on their significant similarity to the N-terminal ribosomal S6 kinases (RSKs). Human MSK1 and MSK2 are 63–75% identical to each other, and present significant homology to RSKs (approx. 40% similarity) (Deak et al. 1998). MSK1 and MSK2 are activated through MAPK/ERKs signalling in response to mitotic agents such as epidermal growth factor (EGF) and phorbol ester (TPA, 12-O-tetradecanoylphorbol-13-acetate); or through the MAPK/p38 pathway in response to stress stimuli such as UV radiation, oxidative and chemical stress (Deak et al. 1998). Both MSK1 and MSK2 are ubiquitously expressed across a variety of tissues, including heart, brain, placenta, lung, liver kidney and pancreatic tissue, with predominant expression observed in brain, placenta and skeletal muscle (Deak et al. 1998).

Many transcription factors have been identified as substrates of MSKs, and phosphorylation by MSKs can enhance their activity. MSK1/2 can phosphorylate cAMP response element-binding (CREB) protein and activating transcription factor 1 (ATF1) in response to mitogens and stress; the phosphorylation of CREB at Ser133 can regulate the transcription of several immediate early (IE) genes, including c-fos, JunB and Egr1 (Wiggin et al. 2002). MSKs contribute to regulation of histone H3, a component of the nucleosome, and HMGN1 (HMG-14), a chromatin-associated protein. It has been shown that stress- and mitogen-stimulated phosphorylation of histone H3 and HMGN1 in fibroblasts is compromised in MSK1^−/−^MSK2^−/−^ mice (Soloaga et al. 2003). The physiological function(s) of MSKs remains to be completely determined; however, it has been suggested that MSK1/2 may have a role in regulating the immune system and neuronal function [reviewed in (Vermeulen et al. 2009)].

Recent studies have suggested that MSK1 may be important in the regulation of breast cancer cell progression, playing a role in steroid-hormone induced breast cancer cell proliferation (Reyes et al. 2014). Depletion of MSK1 has been shown to inhibit oestrogen (E2) or progesterone-induced breast cancer cell (T-47D) proliferation and tumour growth in hormone-dependent breast cancer xenografts (Reyes et al. 2014). Furthermore, MSK1 participates in G1-S phase transition and regulates cell cycle-associated gene expression in response to hormones, evidenced by the recruitment of MSK1 to specific PR-binding sites in chromatin (Reyes et al. 2014). Breast tumour-associated osteoblast (TAOB)-derived CXCL5 induced cell migration, and invasion of the breast cancer cell lines, MCF-7 and MDA-MB-231, is associated with increased MSK1 activation (Hsu et al. 2013). In T-47D breast cancer cells, alcohol exposure can cause increased expression and activation of proto-oncogene tyrosine-protein kinase (ROS1), which induces MSK1 activation through the MAPK/ERKs pathway (Lee et al. 2013).

The aim of the current study was to assess MSK1 protein expression in a large cohort of breast cancer tumour specimens, evaluating its prognostic significance.

## Methods

### Clinical samples

This immunohistochemical-based study was performed using a cohort of early stage breast cancer patients (*n* = 1902) treated at Nottingham University Hospitals, with long-term follow-up, between 1986 and 1998. Information on clinical history, outcome and tumour characteristics was collected and assessed in a standardised manner, including age at diagnosis, tumour size, histologic stage and grade, Nottingham prognostic index (NPI), lymphovascular invasion (LVI), oestrogen receptor (ER), progesterone receptor (PR) and human epidermal growth factor receptor 2 (HER2) status. ER, PR and HER2 status were available for this cohort and have been described previously (Abdel-Fatah et al. 2010). HER2 positivity expression was determined by immunohistochemistry (IHC) with fluorescence in situ hybridisation (FISH), and FISH was performed for the cases with IHC scored as 2+. Basal-like phenotype was defined as the expression of cytokeratin (CK)-5/6 and/or CK-14 expression in 10% or more of invasive tumour cells, irrespective of ER, PR or HER2 status (Rakha et al. 2006). The median age of the patients was 55 years (ranging from 18 to 72), and 63.2% (1203 of 1902) of patients had stage I disease. Breast cancer-specific survival was defined as the time interval (in months) from the date of primary surgery to death resultant from breast cancer. The median follow-up time was 177 months (ranging from 1 to 308 months). Patients were managed under a uniform protocol, where all underwent wide local excision (*n* = 819, 43.1%) or mastectomy (*n* = 1067, 56.1%), and approximately half of the patients received radiotherapy (*n* = 1025, 53.9%). Systemic adjuvant treatment was given based on ER, menopausal status and NPI values. Patients with ER-positive disease were chosen for hormone therapy (*n* = 674, 35.4%). Patients with ER negative or premenopausal status were chosen for CMF chemotherapy (cyclophosphamide, methotrexate and 5-fluorouracil, *n* = 320, 16.8%), if they had an NPI value of 3.4 or above, whereas patients who had an NPI value less than 3.4 did not receive adjuvant chemotherapy. This study is reported in accordance with REMARK criteria (McShane et al. 2005). Nottingham Research Ethics Committee 2 approved the project under “Development of a molecular genetic classification of breast cancer R&D “(No. 03HI01 REC Ref.C202313)”. The clinicopathologic variables of the patient cohort are shown in Table 1.

**Table 1:** Clinicopathologic variables of breast cancer patient cohort

Variables	No. (%)
Age (mean ± SD, years)	54.25 (± 9.77)
≤ 40 years	165 (8.7%)
> 40 years	1736 (91.3%)
ND	1 (0.1%)
Tumour size (mm)	2.06 ± 1.14
≤ 20 mm	1185 (62.3%)
> 20 mm	708 (37.2%)
ND	9 (0.5%)
Tumour stage	
I	1203 (63.2%)
II	531 (27.9%)
III	160 (8.4%)
ND	8 (0.4%)
Tumour grade	346 (18.2%)
I	
II	632 (33.2%)
III	915 (48.1%)
ND	9 (0.5%)
NPI	4.16 ± 1.18
≤ 3.4	619 (32.5%)
3.41–5.4	948 (49.8%)
> 5.4	324 (17.0%)
ND	11 (0.6%)
Lymphovascular invasion	
Positive	492 (25.9%)
Negative	1070 (56.3%)
ND	340 (17.9%)
Operation type	
Mastectomy	1067 (56.1%)
WLE lumpectomy	819 (43.1%)
ND	16 (0.8%)
ER status	
Positive	1370 (72.0%)
Negative	476 (25.0%)
ND	57 (3.0%)
PR status	
Positive	1035 (54.4%)
Negative	739 (38.9%)
ND	128 (6.7%)
HER2 status	
Positive	243 (12.8%)
Negative	1602 (84.2%)
ND	57 (3.0%)
Basal status	
Positive	368 (19.3%)
Negative	1390 (73.1%)
ND	144 (7.6%)
Triple negative status	
Positive	315 (16.6%)
Negative	1516 (79.7%)
ND	71 (3.7%)
Breast cancer-specific survival	
Alive	1064 (55.9%)
Dead	505 (26.6%)
ND	333 (17.5%)
Recurrence	
Present	752 (39.5%)
Not present	1103 (58.0%)
ND	47 (2.5%)
Distant metastasis	
Present	579 (30.4%)
Not present	1310 (68.9%)

Continuous data are shown as mean ± standard deviation (SD)

*NPI* Nottingham prognostic value; *WLE* wide local excision; *ER* oestrogen receptor; *PR* progesterone receptor; *HER2* human epidermal growth factor receptor 2; *ND* not determined

### TMA construction and immunohistochemistry

MSK1 protein expression was investigated using freshly cut 4 µm sections from tissue microarrays and assessed by immunohistochemistry. A single 0.6 mm tissue core was used for each patient with the core being taken from a representative tumour area as assessed by a specialist breast cancer histopathologist, as described previously (Abd El-Rehim et al. 2005). A range of antibodies were assessed for suitability with primary antibody specificity being confirmed by Western blotting, using a range of breast cancer cell line lysates, prior to immunohistochemistry (data not shown). TMA sections were dewaxed in xylene and rehydrated in ethanol, followed by antigen retrieval, in 0.01 mol/L sodium citrate buffer (pH6.0), using a microwave, 750W for 10 min then 450W for 10 min. Staining was achieved using a Novolink Polymer Detection System (Leica, Denmark) according to the manufacturers’ instructions. Briefly, endogenous peroxidase activity was neutralised with Peroxidase Block reagent for 5 min at room temperature, followed by Protein Block reagent for 5 min at room temperature, to minimise non-specific interactions of the subsequent detection reagents. Primary antibody (anti-MSK1, 1: 200, Bethyl Laboratories, USA, 9252) was diluted in Bond Primary Antibody Diluent (Leica, Denmark) and applied to the tissue for 1 h at room temperature. Immunohistochemical reactions were visualised with 3, 3′-diaminobenzidine and counterstained with haematoxylin, dehydrated and fixed in xylene followed by mounting with DPX. Positive controls constituted early stage breast tumour composite sections of varying grades, used in initial optimisations, and negative control omitted primary antibody.

Staining was assessed at 200× magnification following high-resolution scanning (Nanozoomer Digital Pathology Scanner, Hamamatsu Photonics). As no cytoplasmic staining was observed, only nuclear staining intensity was assessed. The staining intensity was categorised as: none (0), weak (1), medium (2) and strong (3). The immunohistochemistry H-scores were calculated by multiplying the percentage of positive tumour cell nuclei by the staining intensity, giving rise to a score ranging between 0 and 300. The immunohistochemistry methodology was described previously (Woolston et al. 2011a, b). 30% of cases were scored by a second independent assessor, blind to survival endpoints, clinicopathological variables, and other assessors H-scores. Good concordance was demonstrated between both scorers (single measure intraclass correlation coefficient was 0.881). X-tile software was used to generate a non-biased cut point that was used to dichotomise immunohistochemical scores (Camp et al. 2004).

### Statistical analysis

Pearson’s chi-squared test of association (*χ*^2^) was used to examine the relationship between categorised protein expression and clinicopathologic factors, or Fisher’s exact test if a cell count in a 2 × 2 table was less than 5. Kaplan–Meier survival curves were plotted and significance was determined using the Log-rank test. The Cox proportional hazards regression model was applied in multivariate survival analysis. All differences were considered statistically significant at the level of *P* < 0.05. Statistical analysis was performed using SPSS 22.0 software.

## Results

### Immunohistochemical staining

**Fig. 1:**
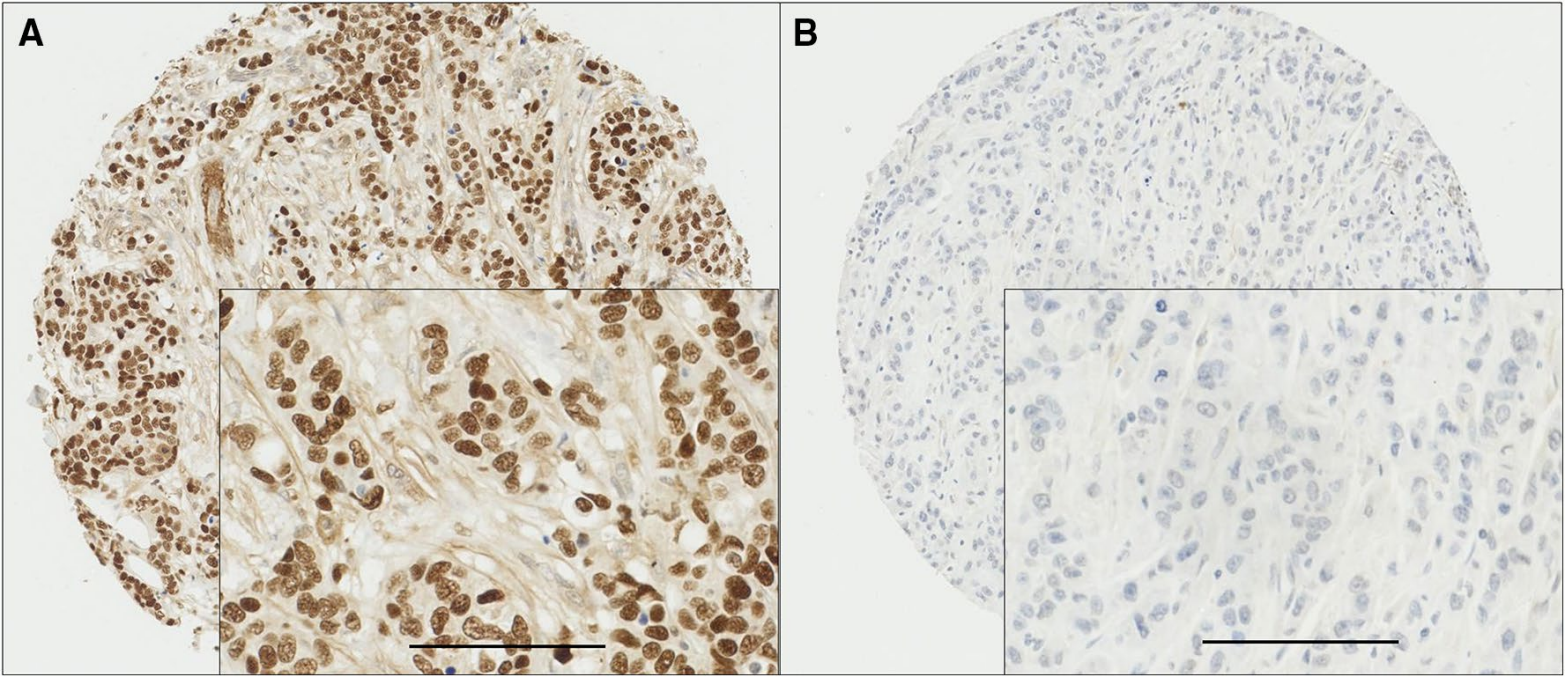
Representative photomicrographs of high and low MSK1 expression. Panel A (high expression) and panel B (low expression) at ×10 magnification with ×20 magnification inset panel and scale bar representing 100 µm

MSK1 demonstrated diffuse nuclear staining with some heterogeneity in intensity which varied from weak to intense. No cytoplasmic staining was observed. Areas of ductal carcinoma in situ (DCIS) showed variable staining intensity in some cases. Only cases of invasive cancer were scored. Typical staining patterns are shown in Fig. 1. TMA cores with insufficient tumour cells (< 15%) were not considered in the analysis. A total number of 1270 cases with informative cores were assessed. MSK1 staining had a median H-score of 190 ± 83 and ranged from 0 to 300. X-tile software was used to generate a cut point best associated with outcome. This indicated an H-score of 115, with 364 cases (28.7%) having low expression and 906 cases (71.3%) having high expression.

### Relationships with clinicopathologic variables

High MSK1 expression was significantly associated with patients over 40 years (*χ*^2^ = 8.283, *df* = 1, *P* = 0.004), lower tumour grade (*χ*^2^ = 34.505, *df* = 2, *P* < 0.001), lower NPI value (*χ*^2^ = 9.804, *df* = 2, *P* = 0.007), ER-positive tumours (*χ*^2^ = 50.186, *df* = 1, *P* < 0.001) and PR-positive tumours (*χ*^2^ = 41.147, *df* = 1, *P* < 0.001). Low MSK1 expression was significantly associated with the presence of triple-negative (*χ*^2^ = 56.406, *df* = 1, *P* < 0.001) and basal-like tumours (*χ*^2^ = 13.850, *df* = 1, *P* < 0.001). The associations between protein expression and clinicopathologic variables are shown in Table 2.

**Table 2:** Associations between MSK1 protein expression and clinicopathologic variables

Variables	MSK1 (*n* = 1270)		
	Low expression	High expression	*P*-value
Age (years)			
≤ 40 years	49 (3.9%)	74 (5.8%)	**0.004**
> 40 years	315 (24.8%)	831 (65.5%)	
Tumour size (mm)			
≤ 20 mm	207 (16.4%)	545 (43.0%)	0.275
> 20 mm	156 (12.3%)	358 (28.3%)	
Tumour stage			
I	227 (17.9%)	540 (42.7%)	0.544
II	105 (8.3%)	275 (21.7%)	
III	30 (2.4%)	89 (7.0%)	
Tumour grade			
I	38 (3.0%)	169 (13.3%)	< **0.001**
II	96 (7.6%)	326 (25.8%)	
III	229 (18.1%)	408 (32.2%)	
Nottingham prognostic index			
≤ 3.4	85 (6.7%)	290 (22.9%)	**0.007**
3.41–5.4	198 (15.7%)	451 (35.7%)	
> 5.4	79 (6.3%)	161 (12.7%)	
Lymphovascular invasion			
Positive	107 (10.1%)	248 (23.4%)	0.263
Negative	190 (17.9%)	517 (48.7%)	
Operation type			
Mastectomy	213 (16.9%)	520 (41.3%)	0.682
WLE Lumpectomy	147 (11.7%)	378 (30.0%)	
ER status			
Positive	205 (16.7%)	704 (57.2%)	< **0.001**
Negative	139 (11.3%)	183 (14.9%)	
PR status			
Positive	147 (12.3%)	538 (45.0%)	< **0.001**
Negative	196 (16.4%)	314 (26.3%)	
HER2 status			
Positive	49 (3.9%)	125 (10.1%)	0.894
Negative	306 (24.6%)	762 (61.4%)	
Basal-like status			
Positive	96 (8.1%)	153 (13.0%)	< **0.001**
Negative	247 (20.9%)	685 (58.0%)	
Triple-negative status			
Positive	104 (8.5%)	109 (8.9%)	< **0.001**
Negative	237 (19.4%)	774 (63.2%)	
Breast cancer-specific survival			
Alive	577 (41.7%)	342 (24.7%)	**0.01**
Dead	259 (18.7%)	207 (14.9%)	
Recurrence			
Present	167 (13.5%)	362 (29.3%)	**0.013**
Not present	178 (14.4%)	530 (42.8%)	
Distant metastasis			
Present	138 (11.0%)	267 (21.2%)	**0.002**
Not present	220 (17.5%)	635 (50.4%)	

Correlations between MSK1 protein expression and clinicopathologic variables was assessed using Pearson’s Ch-square test of association (*χ*^2^) or Fisher’s exact test if in a 2 × 2 tables and cell count was less than 5. Significant *P* values are indicated by bold font

*ER* oestrogen receptor, *PR* progesterone receptor, *HER2* human epidermal growth factor receptor 2

### Relationships with clinical outcome

**Fig. 2:**
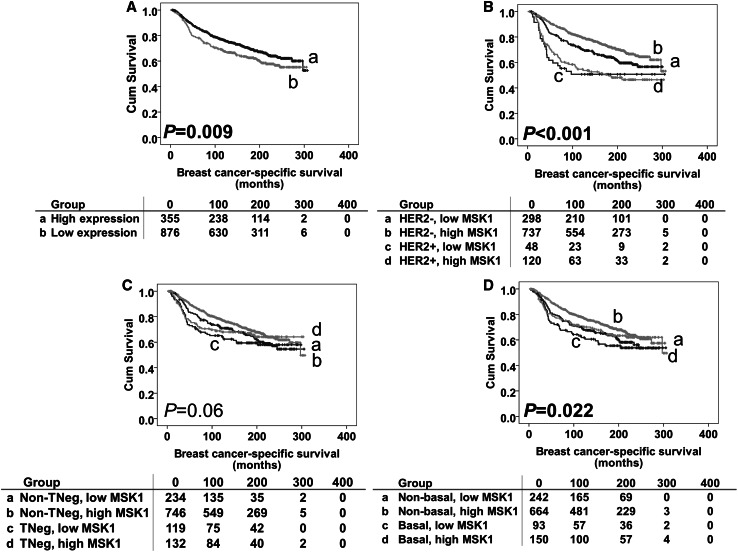
Kaplan–Meier survival curves for breast cancer-specific survival based on MSK1 expression. Significance was determined using the log-rank test. Panel A: high MSK1 (**a**) and low MSK1 (**b**) expression in total patient cohort. Panel B: HER2-negative disease with low MSK1 expression (**a**); HER2-negative disease with low MSK1 expression (**b**); HER2-positive disease with high MSK1 expression (**c**); HER2-positive disease with high MSK1 expression (**d**). Panel C: non-triple-negative disease with low MSK1 expression (**a**); non-triple-negative disease with low MSK1 expression (**b**); triple-negative disease with high MSK1 expression (**c**); triple-negative disease with high MSK1 expression (**d**). Panel D: non-basal-like disease with low MSK1 expression (**a**); non-basal-like disease with low MSK1 expression (**b**); basal-like disease with high MSK1 expression (**c**); basal-like disease with high MSK1 expression (**d**). The numbers below the survival curves are the number of patients at risk at the specified month

Univariable survival analysis showed that low MSK1 expression was, in the whole patient cohort, significantly associated with worse breast cancer-specific survival (*P* = 0.009) (Fig. 2). Multivariate analyses including potential confounding factors, namely age at diagnosis, size, histologic stage, grade, NPI value, lymphovascular invasion, ER, PR and HER2 status (all variables with individual Kaplan–Meier statistics of *P* < 0.05) were carried out to determine MSK1’s independent prognostic value. MSK1 expression was not an independent indicator of breast cancer-specific survival [hazard radio (HR) = 0.899, 95% confidence interval (CI) 0.696–1.161; *P* = 0.415].

Low MSK1 expression was also significantly associated with the development of distant metastasis (*χ*^2^ = 13.850, *df* = 1, *P* < 0.001), recurrence (including local/regional recurrence and distant metastasis) (*χ*^2^ = 6.220, *df* = 1, *P* = 0.013) and breast cancer-related death (*χ*^2^ = 6.711, *df* = 1, *P* = 0.01).

**Fig. 3:**
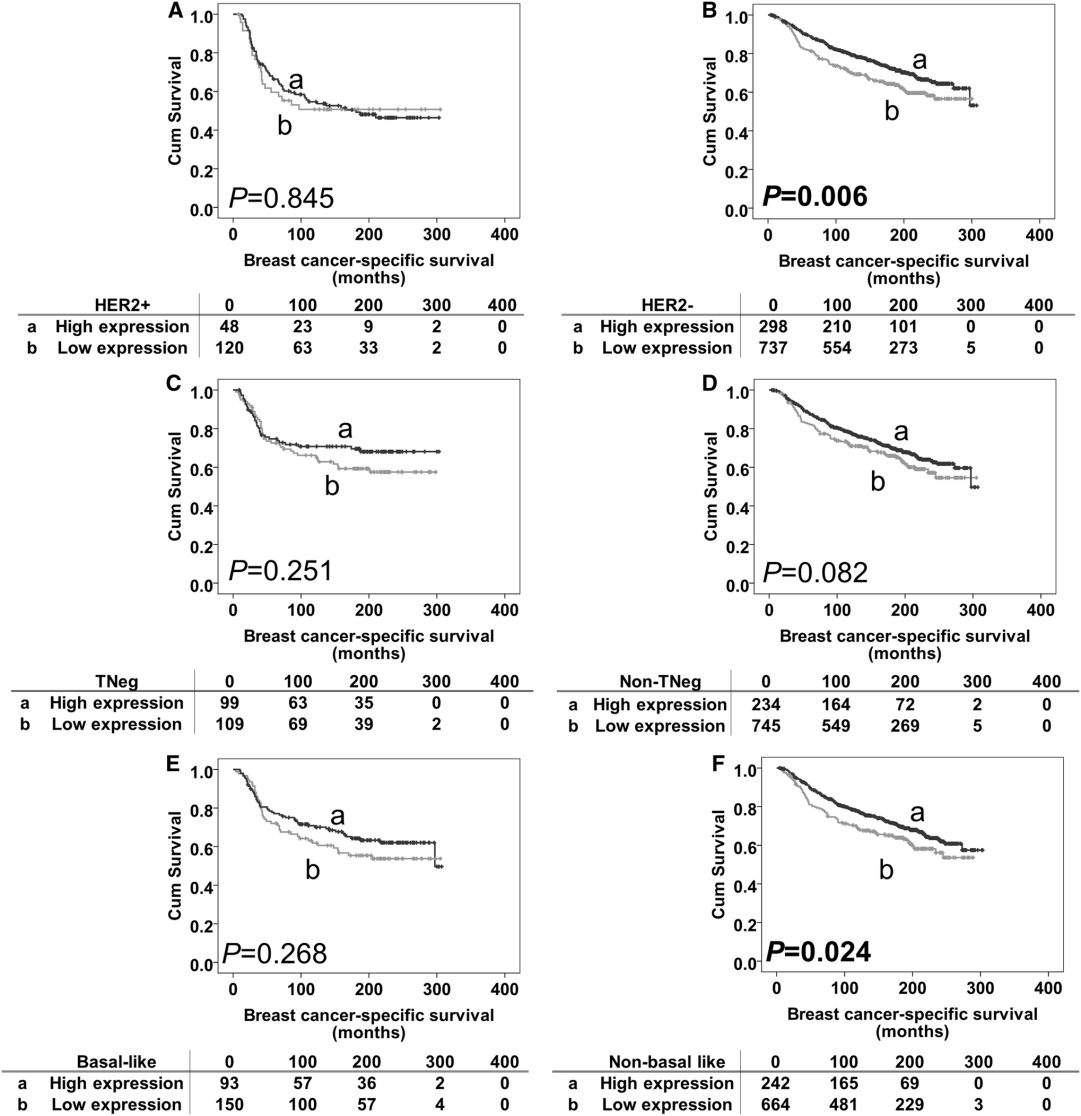
Kaplan–Meier survival curves for breast cancer-specific survival based on MSK1 expression in subgroups. Panel A: subgroup analysis of HER2-positive disease with high MSK1 expression (**a**) and low MSK1 expression (**b**). Panel B: subgroup analysis of HER2-negative disease with high MSK1 expression (**a**) and low MSK1 expression (**b**). Panel C: subgroup analysis of triple-negative disease with high MSK1 expression (**a**) and low MSK1 expression (b). Panel D: subgroup analysis of non-triple-negative disease with high MSK1 expression (**a**) and low MSK1 expression (**b**). Panel E: subgroup analysis of basal-like disease with high MSK1 expression (**a**) and low MSK1 expression (**b**). Panel F: subgroup analysis of non-basal-like disease with high MSK1 expression (**a**) and low MSK1 expression (**b**). The numbers below the survival curves are the number of patients at risk at the specified month

The prognostic significance of MSK1 protein expression was further assessed in different molecular phenotypes of breast tumours. As shown in Table 1, 243 (12.8%), patients had HER2 + phenotype tumours, 315 (16.6%) patients had triple-negative phenotype tumours, and 368 (19.3%) patients had basal-like phenotype (as defined in the “methods” section) tumours. MSK1 expression was significant in both HER2 and basal-like groups (association with outcome breast cancer-specific survival) (*P* < 0.001 and *P* = 0.022, respectively, Fig. 2b, d). This was investigated further in the individual subgroups; low MSK1 expression was associated with adverse breast cancer-specific survival in HER2-negative patients and patients with non-basal like disease (*P* = 0.006 and *P* = 0.024, respectively, Fig. 3b, f). No significant association was found in other individual subgroups (Fig. 3a, c–e). However, when including the previous confounding factors in multivariate analysis, MSK1 lost its independent prognostic value for breast cancer-specific survival in both the HER2-negative (hazard ratio = 0.860, 95% confidence interval 0.649–1.141; *P* = 0.295) and non-basal like subgroup (hazard ratio = 0.938, 95% confidence interval 0.695–1.266; *P* = 0.674).

## Discussion

This is the first study to describe the expression of total MSK1 protein in breast cancer patient tumour samples and to evaluate its prognostic significance. Low MSK1 expression was significantly associated with improved breast cancer-specific survival (*P* = 0.009); however, this was not significant in multivariate analysis.

High MSK1 expression was significantly associated with older patient age, lower tumour grade, lower NPI values, ER-positive and PR-positive tumours, and clinicopathological variables indicative of an improved prognosis. In agreement with this, low MSK1 expression was also found to be associated with the presence of distant metastasis, recurrence and death due to breast cancer, as well as the presence of triple-negative and basal-like disease. As MSK1 expression was associated with triple-negative and basal-like disease, the prognostic capability of MSK1 expression in these patient sub-groups was assessed. Patients with HER2 negative or non-basal like diseases had a significantly worse prognosis if the tumour expressed low levels of MSK1.

Phosphorylated MSK1 has been shown to be significantly overexpressed in chronic UV-exposed murine skin, human squamous cell carcinoma (SCC) and poorly differentiated nasopharyngeal carcinoma compared with their normal tissue counterparts (Yao et al. 2014; Li et al. 2015). In the current study, total MSK1 expression, rather than phosphorylated MSK1, was assessed. Total MSK1 expression may not be indicative of MSK1 activity within cells; therefore, we cannot infer activity in this study. As mentioned before, many factors are substrates of MSKs and can be phosphorylated by MSK, such as CREB, ATF1, c-fos and histone H3 (Wiggin et al. 2002; Soloaga et al. 2003). Antibodies detecting specific MSK1 substrates might be a reflection of MSK1 activity in human breast tumour tissue. Low expression of H3K27 (with loss of trimethylation of histone H3 at Lysine 27) was also associated with adverse overall survival in breast (*n* = 142), ovarian (*n* = 255) and pancreatic (*n* = 165) cancer patients (Wei et al. 2008). In addition, MSK1 activity in vitro (human embryonic kidney 293 cells) is tightly regulated by at least six phosphorylation sites; the mutation of some sites leads to decreased MSK1 activity or inhibition of other phosphorylation sites in MSK1 in cells (McCOY et al. 2005). It would be of interest to determine MSK1 expression in human tumour tissue by applying MSK1 antibodies with different phosphorylation sites and to explore whether MSK1 expression is regulated by a specific phosphorylation site.

Although MSK1 and MSK2 share similarity, they are different complexes and exert differing effects. Interestingly, differential roles of MSK1 and MSK2 in breast cancer cells following UV-radiation have been described. UV radiation has been shown to induce activation of the NF-κB p65 subunit in MDA-MB-231 cells, which is mainly dependent on MSK2 rather than MSK1. The depletion of MSK2 was found to reduce cell viability following UV radiation, suggesting that MSK2-mediated NF-κB activation is involved in regulating cell survival in MDA-MB-231 cells (Jacks and Koch 2010). Recently, MSK1 and MSK2 were also found to be involved in the regulation of phorbol ester-induced activation of trefoil factor 1 (TFF1), a breast cancer marker, in MCF-7 cells (Khan et al. 2013). It would be of interest to examine the potential differential roles of MSK1 and MSK2 in breast cancer cell progression, as well as their prognostic significance.

## Conclusions

The current study demonstrates that low MSK-1 expression is significantly associated with adverse breast cancer-specific survival. Low expression seems to play a more important role in certain patient sub-groups, such as those with HER2 + or basal-like phenotype disease. This is the first report to describe the potential prognostic significance of MSK1 expression in breast cancer patients; although not an independent marker of outcome, we believe such findings and significant associations with well-established negative prognostic factors (low expression associating with younger age, higher tumour grade, higher Nottingham Prognostic Index, negative hormone receptor status, shorter time to distant metastasis, and recurrence, and triple-negative/basal-like status) warrant further examination and validation in independent patient cohorts.
